# Modeling Incoherent Discourse in Non-Affective Psychosis

**DOI:** 10.3389/fpsyt.2020.00846

**Published:** 2020-08-19

**Authors:** Sandra A. Just, Erik Haegert, Nora Kořánová, Anna-Lena Bröcker, Ivan Nenchev, Jakob Funcke, Andreas Heinz, Felix Bermpohl, Manfred Stede, Christiane Montag

**Affiliations:** ^1^Department of Psychiatry and Psychotherapy, Campus Charité Mitte (Psychiatric University Clinic at St. Hedwig Hospital), Charité-Universitätsmedizin Berlin, Corporate Member of Freie Universität Berlin, Humboldt-Universität zu Berlin, and Berlin Institute of Health, Berlin, Germany; ^2^Applied Computational Linguistics, UFS Cognitive Science, University of Potsdam, Potsdam, Germany

**Keywords:** automated analysis, schizophrenia, psychosis, speech, coherence, thought disorder

## Abstract

**Background:**

Computational linguistic methodology allows quantification of speech abnormalities in non-affective psychosis. For this patient group, incoherent speech has long been described as a symptom of formal thought disorder. Our study is an interdisciplinary attempt at developing a model of incoherence in non-affective psychosis, informed by computational linguistic methodology as well as psychiatric research, which both conceptualize incoherence as associative loosening. The primary aim of this pilot study was methodological: to validate the model against clinical data and reduce bias in automated coherence analysis.

**Methods:**

Speech samples were obtained from patients with a diagnosis of schizophrenia or schizoaffective disorder, who were divided into two groups of n = 20 subjects each, based on different clinical ratings of positive formal thought disorder, and n = 20 healthy control subjects.

**Results:**

Coherence metrics that were automatically derived from interview transcripts significantly predicted clinical ratings of thought disorder. Significant results from multinomial regression analysis revealed that group membership (controls vs. patients with vs. without formal thought disorder) could be predicted based on automated coherence analysis when bias was considered. Further improvement of the regression model was reached by including variables that psychiatric research has shown to inform clinical diagnostics of positive formal thought disorder.

**Conclusions:**

Automated coherence analysis may capture different features of incoherent speech than clinical ratings of formal thought disorder. Models of incoherence in non-affective psychosis should include automatically derived coherence metrics as well as lexical and syntactic features that influence the comprehensibility of speech.

## Introduction

Speech impairments in non-affective psychosis (NAP) can impede communication up to “discourse failure” (1). Impairments comprise difficulties with structural aspects (2–4), the pragmatic use of language (5, 6) as well as cohesion (7–10) and semantic coherence (11–15). Incoherence is a particularly impairing symptom of schizophrenia (16–18). Clinicians usually evaluate incoherent speech by relying on standardized rating scales [e.g. (19)].

The linguistic definition of coherence regards the deeper semantic meaning of speech (often modeled through so-called coherence relations holding among the propositions being expressed) – in contrast to cohesion, which is an aspect of the text surface [(20), p. 25]. Cohesive markers establish syntactic connections between text parts (temporal, causal, referential, etc.) which can still lack semantic coherence, i.e. meaningfulness [(20), p. 33]. The following example contains patient’s speech that is partially incoherent, but still relatively rich of cohesive markers (in italics):

“*When* you are under tension, *then* you cannot feel joy. *When* you are relaxed, *when* the tension somehow … *then* you can feel joy. *And to that effect, that* I am under tension, I cannot feel joy that you, *namely*, look stupid. *Therefore*, I *already* have conned you. I, do you seriously want to tell me? *So*, joy.”

The conceptualization of incoherent speech by Andreasen (19) is very influential in clinical psychiatric research. She describes incoherent speech as one sign of formal thought disorder (FTD), which can occur in numerous mental disorders, albeit predominantly in psychosis (21–23). The Scale for the Assessment of Positive Symptoms (SAPS) defines incoherent speech as loss of associations within sentences, which can result in incomprehensible “schizophasia” or “word salad” (17). Andreasen (19) connects incoherence to other manifestations of positive FTD such as tangentiality (i.e. irrelevant responses to questions), derailment (i.e. loss of associations between larger units of speech), illogical, and indirect speech. Incoherent speech is not present in all NAP patients (18, 24) and can vary depending on the stage of illness and the presence and severity of other symptoms (21, 25, 26).

Ditman and Kuperberg (16) link incoherent speech in NAP to problems in “integrating meaning across clauses” (p. 7) that can lead to a lower similarity in meaning between sentences. This idea invites for automated coherence analysis in computational linguistics because it models coherence as a similarity or overlap between concepts – a speaker is expected to adhere to an established topic to a certain degree at any given stage of the conversation to be considered coherent. Thus, computational linguistics and psychiatry both define incoherent discourse as a decreased semantic similarity between discourse units. Latent Semantic Analysis [LSA; (27)] was the first automated coherence measure used in schizophrenia research (11, 12) (see Ratana, Sharifzadeh, Krishnan, and Pang (28) for a review of automated speech analysis in NAP). Elvevåg et al. (11) were able to differentiate NAP patients from controls based on LSA scores and reported significant correlations between LSA scores and ratings of formal thought disorder. For the analysis of free speech, Elvevåg et al. (11) focused on tangentiality rather than incoherence, measuring similarity between question and response. Bedi et al. (12) were able to predict psychosis development in high-risk individuals with a model that defines coherence as semantic similarity between pairs of adjacent sentences. The LSA-based coherence measure combined with syntactic markers (maximum phrase length, use of determiners) was superior to clinical ratings indicating that automatically derived coherence metrics may represent a highly sensitive measure to detect even sub-clinical incoherence. This was supported by Corcoran et al. (15) who were similarly able to predict psychosis onset in high-risk individuals by combining automatically derived coherence measures with syntactic markers like possessive pronouns. Iter et al. (13) recently improved LSA-based models (11, 12) by preprocessing their data set, filtering stop words and fillers and using modern word and sentence embeddings that were shown to outperform LSA (29, 30). Word embeddings were also used by Rezaii, Walker, and Wolff (31) who were able to predict psychosis onset in high-risk individuals based on word embeddings and participants’ choice of words, and Bar et al. (32) who found that NAP patients adhered less to a topic throughout a conversation than controls. Word embeddings such as Global Vectors for Word Representation [GloVe; (33)] create a vector space based on a large number of texts. In this space, each word is assigned a corresponding vector, and proximity between word vectors represents semantic similarity. Thus, like LSA, GloVe also uses global cooccurrence counts. However, in contrast to LSA, GloVe uses weighted cooccurrences, i.e. vectors can be scaled according to the informativity of the corresponding words using a range of weighting schemes such as Term Frequency-Inverse Document Frequency [TF-IDF; (34)]. Sentence embeddings are represented by the mean vector of their underlying word embeddings. Semantic similarity is defined as “the cosine of the angle […] between two vectors […], with greater cosine values indicating greater degrees of similarity” (11).

In a preliminary study (14), models of coherence by Elvevåg et al. (11) and Bedi et al. (12), with improvements by Iter et al. (13), were transferred to German language and applied to a NAP patient sample. The *Incoherence Model* by Bedi et al. (12), i.e. a measure of coherence based on semantic similarity between adjacent sentences, combined with GloVe and the TF-IDF embeddings yielded significant group differences: lower automatically derived coherence metrics were found for patients with positive FTD than for those without positive FTD, and automatically derived coherence metrics of healthy controls (HC) were higher than those of patients (14). However, potential bias in the model and potential relationships between incoherence and other relevant variables (referential abnormalities, neologisms) had to be discussed.

The present methodological study aims to 1) further validate the *Incoherence Model* against clinical data, to 2) address bias in the model, and to 3) improve its predictive value. For the second goal, we consider that measuring incoherence in NAP via concept overlap can be biased by exclusion of relevant words that do not appear in the reference corpus, such as neologisms, and by perseveration. Perseveration presents a problem as automated coherence analysis is based on the similarity between sets of keywords, without actually accounting for whether the speech is intelligible, and is therefore especially sensitive to bias by inadequate repetition (13). For the third goal, we address that clinical ratings of incoherent discourse may not only be informed by concept similarity but also by comprehensibility of discourse. We therefore introduce additional measures by taking into consideration that comprehensibility of NAP patients’ utterances has been shown to be impaired by abnormal use of referents (1, 16, 35–37) and neologisms (38). Moreover, we analyze the cohesive structure of speech, which is a necessary, albeit not sufficient characteristic of coherent speech (20, 35) and has been shown to improve coherence analysis (12).

In summary, this pilot study aims to examine the following hypotheses:

1. Automatically derived coherence metrics match clinical ratings of positive formal thought disorder.
2. Group membership (healthy controls, patients with/without positive FTD) is predicted by automatically derived coherence metrics when bias by inadequate repetition is controlled for.
3. Modeling disordered thought in NAP can be improved by integrating other quantifiable coherence measures like a) abnormal use of referential markers, b) number of neologisms, and c) syntactic markers of cohesion.

## Materials and Methods

### Participants

*N* = 60 participants were included (see **Table 1** for characteristics of the sample), which is comparable to sample sizes in prior research dealing with automated speech analysis in NAP samples (11–13, 31). *n* = 12 were patients from the Psychiatric University Clinic at St. Hedwig Hospital Berlin and *n* = 28 patients were recruited from the pool of participants in the MPP-S study (clinical trials ID: NCT02576613). Participants were: (1) inpatients (*n* = 7) or outpatients (*n* = 33) with a diagnosis of schizophrenia (*n* = 33) or schizoaffective disorder (*n* = 7) according to Diagnostic and Statistical Manual of Mental Disorders, Fourth Edition, Text Revision (DSM-IV-TR), confirmed by trained clinicians; (2) showed native proficiency in German language; (3) had no organic mental disorder or relevant severe somatic disease; (4) no active substance dependence. Healthy control subjects (*n* = 20) were recruited from the local community and screened by experienced clinicians with the Mini-International Neuropsychiatric Interview (M.I.N.I.) (39). Half of all participants were also included in a preliminary study (14). All participants provided written informed consent. The study was approved by the local ethics’ committee.

**Table 1:** Characteristics of the sample.

	NAP with positive FTD (*n* = 20)	NAP without positive FTD (*n* = 20)	HC (*n* = 20)	Statistics	*p*-value
Age (years)	45.7 (11.91) ^†^	41.9 (10.87)	43.9 (13.29)	*F* ^a^ = .5	.61
Sex (male)	*n* = 15	*n* = 7	*n* = 11	*χ²* ^b^ = 6.47	.04
Verbal IQ	103.6 (14.86)	106.1 (12.61)	103.25 (7.62)	Welch’s *F* ^c^ = .37	.69
Inpatients	*n* = 7	*n* = 0		*χ²* ^d^ = 8.49	.004
F20.0	*n* = 16	*n* = 17		*χ²* = .17	.68
F25.0	*n* = 4	*n* = 3		*χ²* = .17	.68
Antipsychotic medication	*n* = 18	*n* = 20		*χ²* = 2.11	.15
CGI	5.2 (1.36)	3.65 (1.31)		*t* ^e^ = −3.67	.001
Duration of illness (years)	17.25 (12.03)	14.35 (9.91)		*t* = -.83	.41
SAPS					
positive FTD	2.8 (.7)	.35 (.59)		*t* = −12.03	<.001
Incoherence	1.4 (1.55)	.05 (.22)		*t* = −4.25	<.001
Tangentiality	2.6 (.82)	.05 (.22)		*t* = −13.41	<.001
Derailment	2.25 (1.29)	.15 (.67)		*t* = −6.45	<.001
Illogicality	1.5 (1.54)	.05 (.22)		*t* = −4.17	<.001
Circumstantiality	1.7 (1.66)	.55 (.89)		*t* = −2.74	.009
Pressured speech	2.15 (1.6)	.4 (.88)		*t* = −4.29	<.001
Distractibility	1.25 (1.29)	.1 (.45)		*t* = −3.76	.001
Clanging	.6 (1.0)	0		*t* = −2.7	.01
Hallucinations	1.6 (1.67)	1.0 (1.49)		*t* = −1.2	.24
Delusions	2.6 (1.23)	.7 (1.03)		*t* = −5.29	<.001
Bizarre Behavior	1.3 (1.26)	.05 (.22)		*t* = −4.37	<.001
Inappropriate Affect	.85 (1.23)	.05 (.22)		*t* = −2.87	.009
SANS			–		
Flat Affect	1.55 (1.54)	1.3 (1.13)		*t* = -.59	.56
Alogia	1.0 (1.38)	.85 (1.09)		*t* = -.38	.7
Avolition/apathy	1.95 (1.47)	1.55 (1.47)		*t* = -.86	.39
Anhedonia/asociality	2.6 (1.31)	1.95 (1.47)		*t* = −1.48	.15
Attention	.95 (1.13)	.15 (.67)		*t* = −2.66	.012

^†^Mean (SD); group comparisons between healthy controls (HC) and patients with non-affective psychoses (NAP) with and without formal thought disorder (FTD): ^a^ANOVA; ^b^χ²-test; ^c^Welch’s ANOVA; group comparisons between patients with and without FTD: ^d^χ²-test; ^e^t-test for independent samples. CGI: Clinical Global Impression; SANS, Scale for the Assessment of Negative Symptoms; SAPS, Scale for the Assessment of Positive Symptoms.

### Measures

#### Narrative of Emotions Task (NET)

Speech samples for automated analysis were obtained by trained clinicians with a short semi-structured interview, the Narrative of Emotions Task (NET) (40). It includes three questions about four emotions: sadness, fear, anger, and happiness: (1) What does this emotion mean to you? (2) Describe a situation where you felt this emotion. (3) Why do you think you felt this emotion in this situation? The interview is designed to prompt participants to define this range of simple emotions to “assess the richness and coherence with which one explains emotional and social events” (40). Semi-structured interviews have already been used in studies on automated speech analysis in NAP (10, 11). The structured format allows direct comparison between subjects and open questions generate larger samples of free speech. All NET interviews were conducted in German and recorded. They were manually transcribed by the first and third author.

#### Psychopathology

Psychopathology was rated by trained clinicians in the course of a diagnostic interview, using the Scale for the Assessment of Negative Symptoms (SANS) (41) and the Scale for the Assessment of Positive Symptoms (SAPS) (19). Both scales have good psychometric properties and have frequently been used in schizophrenia research (42, 43). The patient sample was divided into two groups based on SAPS ratings: the group with positive FTD was defined by SAPS ratings of at least mild (≥ 2) global positive FTD and at least mild incoherence or tangentiality (≥ 2), as those appeared to be most relevant for incoherence analysis.

#### Severity of Illness

The Clinical Global Impression – Severity Scale (CGI) (44) allows trained clinicians to assess the severity of a patient’s illness on a scale from 1 (not at all ill) to 7 (extremely severely ill).

#### Verbal Intelligence

**“**Crystallized” verbal intelligence was assessed with a German vocabulary test, the Wortschatztest (WST) which is often used to estimate the premorbid intelligence level (45), since intelligence has been shown to be correlated with narrative abilities in former research (46, 47).

### Data Analysis

#### Preparation of Data

**Figure 1:**
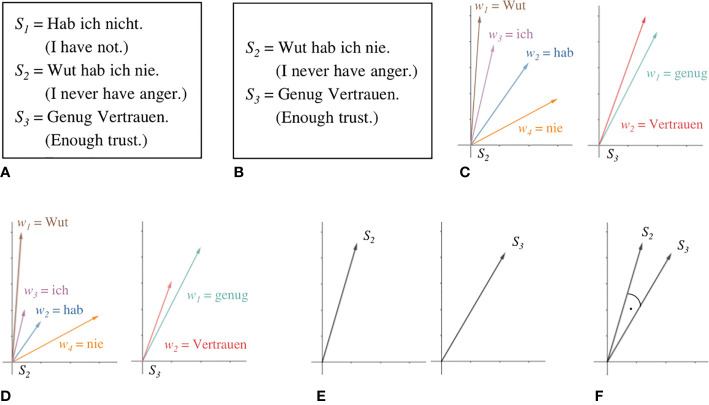
Steps of coherence analysis. Raw data, represented here by three sentences from a transcript **(A)**, is preprocessed by filtering sentences only containing stop words and verbal fillers **(B)**. The meaning of each word is represented as a vector in a semantic space by the GloVe model **(C)**. TF-IDF allows scaling vectors according to their respective semantic contribution **(D)**. Sentence embeddings are calculated as the mean vector of its word embeddings **(E)**. Cosine similarity between adjacent sentences is taken as a measure for level of coherence between them **(F)**.

The data set consisted of 513 min of 60 recorded NET interviews (see **Table 2**). Interview length ranged between 3 and 22 min, with an average length of 8.5 min. The interviewer**’**s speech was left out for complex analysis since it can be reduced to the questions mentioned above. After cleaning transcripts of the interviewer**’**s speech, the raw data set consisted of 46,375 words, ranging from 134 to 2,644 words, with an average of 772.92 words per participant. Examples for raw data are shown in **Figure 1A**. For the coherence models, verbal fillers and sentences only containing stop words were excluded from analysis because they can bias coherence measures (13). An example of this is shown in **Figure 1B**. Words not appearing in the reference corpus for the GloVe model were discarded for this model. Unknown words were saved for later examination, especially for the detection of neologisms. The GloVe model was provided by deepset (48) as open source who trained the model on a German Wikipedia dump.

**Table 2:** Data set.

Word count	Total (*N* = 60)	NAP with positive FTD (*n* = 20)	NAP without positive FTD (*n* = 20)	HC (*n* = 20)	*F*^a^	*p*-value
Raw data	46,375772.92 (493.94)^†^	18,011900.55 (542.81)	10,788539.4 (360.12)	17,576878.8 (496.92)	3.67	.03
GloVe	42,757712.62 (462.57)	16,624831.2 (504.34)	9,772488.6 (331.7)	16,361818.05 (469.81)	3.86	.03

^†^Mean (SD); ^a^ANOVA; group comparisons of healthy controls (HC) and patients with non-affective psychoses (NAP) with and without formal thought disorder (FTD); GloVe data set: transcripts cleaned of sentences only containing stop words, fillers, unknown words.

#### Automated Coherence Analysis

The shift from a taxonomical view on word meaning to one that emphasizes distributional similarities between words has proved to be one of the most fruitful developments of the last decade in natural language processing. Under this perspective, the meaning of a word is captured by the contexts within which it appears. GloVe (33) represents a widely used and effective way to learn semantic representations of words in the form of real-valued and relatively low-dimensional vectors from large amounts of text, called embeddings. The great advantage of such models in comparison to taxonomic resources or high-dimensional, sparse, and orthogonal vector encodings lies in the fact that they provide a natural notion of word similarity. Embeddings of words that appear in similar contexts will not only share some aspect of their meaning but also have vectors that cluster together. The distance between two vectors can hence be interpreted as a measure of the semantic similarity of the words they represent.

**Figure 1** illustrates the steps in coherence analysis. After preprocessing **Figure 1(B)**, each word is assigned a corresponding vector in the GloVe model **Figure 1(C)**. Let *S* be a sentence of length *n* and *w* the embedding of some word.

$$S=\{w_{1},w_{2},w_{3},\text{...}w_{n}\}$$

Following the weighting scheme Term Frequency-Inverse Document Frequency [TF-IDF; (34)], to mitigate the influence of very frequent but semantically poor words, such as articles or prepositions, we scale every word embedding by the ratio of the appearance of their corresponding word in the sentence of interest to the number of documents within a large reference corpus that contain it^1^. For this purpose, we used a lemmatized dump of Wikipedia (2011). Lemmatization aims **“**to remove inflectional endings only and to return the base or dictionary form of a word, which is known as the lemma**”** [p. 32, (49)]. This sharpens the resulting statistic as different morphological forms of one and the same word are all mapped to their respective lemmas. The resulting scalar for very common words will thus be very small, while very uncommon words will be scaled by a number somewhat closer to their actual number of appearances within the sentence under consideration, as shown in **Figure 1D**. We then derive embeddings from entire sentences by using the mean vector of its word embeddings **Figure 1(E)**.

Let *a_i_* be the weight by which *w_i_* is scaled. The corresponding sentence embedding *S_v_* is then computed as follows:

$$Sv=\frac{1}{n}\sum_{i=1}^{n}w_{i}a_{i}$$

Our coherence analysis largely follows the *Incoherence Model* by (12). We take the mean cosine similarity between adjacent sentence embeddings to be a measure of the overall coherence of the entire text. The cosine similarity (represented by the angle in **Figure 1F**) between two vectors A and B is defined by their dot product over the product of their respective magnitudes.

$$\text{sim}=\frac{A\cdot B}{\left\|A\|\|B\right\|}=\frac{\sum_{i=1}^{n}A_{i}B_{i}}{\sqrt{\sum_{i=1}^{n}A_{i}^{2}}\sqrt{\sum_{i=1}^{n}B_{i}^{2}}}$$

Given a text *T* consisting of a sequence of *n* sentences *S* and *S_vi_* representing the sentence embedding of the *i^th^* sentence in *T*, the overall coherence score is computed as follows:

$$\text{Coherence}(T)=\frac{1}{n-1}\sum_{i=1}^{n-1}sim\left(S_{vi},S_{vi+1}\right)$$

#### Repetitions

We developed a script to approximate the problem of bias by perseveration in NAP via controlling for inadequate repetition. It was necessary to differentiate adequate from inadequate repetitions because adequate repetitions, e.g. for emphasis, increase coherence. The script counted repetitions of emotion keywords in responses to question one of the NET interview, where participants were asked to define emotions. The idea behind this approach was that it is, for example, necessary and coherent to use the word “fear” a few times when one is asked to define that emotion. However, a relatively high frequency of emotion keywords was assumed to represent a semantically poor, tautological, and thus inadequate repetition (e.g. “fear is fear is fear”). Thus, a high number of repetitions may represent a failure to develop a more complex and coherent conceptualization of emotion that requires diverging from literal words for emotions (50). Numbers were normalized for word count.

#### Referential Abnormalities

Evaluation of referential abnormalities was based on manual annotation of ambiguous use of pronouns and names throughout the interview transcripts. In contrast to Iter et al. (13), we refrained from using automated coreference resolution which appeared to be relatively error-prone. Both pronouns and names were marked when it remained unclear whom they referred to, as in the following example from a patient’s interview (ambiguous referents in italics):

“For example, we want to do something too often, you know, so when *he* becomes sick from that, you know? Maybe you get little or a little more, like with panic, but so. *He* always says it like that.”

This measure allowed for determining the relative frequency of ambiguous referents per interview.

#### Neologisms

It is worth examining words that were not assigned vectors in the GloVe model. These can either be uncommon or quite specific words (e.g. exacerbation) or neologisms that are more or less intelligible and might impede coherence (e.g. “Rotwut”: “red-rage”; “Leichendurchschauer”: “somebody-who-looks-through-bodies”, presumably for coroner). Thus, unknown words in the GloVe model were screened for neologisms. The mean percentage of words that were characterized as neologisms was calculated per transcript.

#### LIWC

The Linguistic Inquiry Word Count [LIWC; (51)] automatically assesses the relative frequency of certain syntactic features of a text by comparing every single word of a text to a dictionary and mapping it to one or more pre-defined categories. We used the German DE-LIWC2015 dictionary by Meier et al. (52) that comprises more than 18,000 words and more than 80 categories. Following former psycholinguistic research (53, 54), we focused on LIWC categories *conjunctions*, *common adverbs*, *causation*, *differentiation*, and *focus on past*, *present*, or *future* that were connected to additive, temporal and causal markers of cohesion, i.e. “connectives” [p. 198, (53)] that establish relationships between different text parts. LIWC was applied to the raw data set and calculated the percentage of words per transcript in these pre-defined categories. Only categories that differed significantly between groups were included in further analysis (**Table 3**), i.e. *differentiation* cohesive markers (if, when, but, although, etc.).

**Table 3:** Coherence markers, bias and syntactic features: *z*-standardized independent variables.

	NAP with positive FTD	NAP without positive FTD	HC	Statistics	*p*-value
ACM	−.41 (.89) ^†^	.2 (1.2)	.22 (.77)	*F* ^a^ = 2.73	.07
Repetitions	−.32 (.89)	.52 (1.11)	−.2 (.8)	*F* = 4.63	.01
Ambiguous referents	.56 (1.51)	−.2 (.51)	−.36 (.25)	Welch’s *F* ^b^ = 4.11	.03
Neologisms	.59 (1.38)	−.1 (.72)	−.49 (.23)	Welch’s *F* = 8.2	.002
LIWC					
Differentiation	−.42 (.95)	.02 (1.08)	.41 (.82)	*F* = 3.8	.03
Common adverbs	−.09 (1.05)	−.18 (.94)	.27 (1.0)	*F* = 1.12	.33
Conjunctions	−.33 (1.05)	−.01 (1.12)	.34 (.73)	*F* = 2.34	.11
Causation	−.05 (.85)	.19 (1.28)	−.15 (.82)	*F* = .6	.55
Focus past	.13 (.98)	.24 (.94)	−.37 (1.01)	*F* = 2.24	.12
Focus present	−.22 (.98)	.13 (1.03)	.1 (1.0)	*F* = .75	.48
Focus future	.04 (.8)	−.07 (1.32)	.04 (.84)	Welch’s *F* = .06	.95

^†^Mean (SD); ^a^ANOVA; ^b^Welch’s ANOVA; group comparisons of healthy controls (HC) and patients with non-affective psychoses (NAP) with and without formal thought disorder (FTD); ACM, automatically derived coherence metrics; LIWC, Linguistic Inquiry Word Count.

#### Statistics

Statistical analysis was performed using PASW Statistics (version 18.0; SPSS Inc., Chicago, USA). Differences between all three groups were assessed using univariate analysis of variance (ANOVA) or Welch’s ANOVA, depending on the homogeneity of variances. Analyses of differences between the two patient groups were *t*-tests or *χ*²-tests, depending on the level of measurement. For all analyses, the significance level was set at *p* < 0.05. Linear regression analysis was used to predict clinical ratings of FTD with automatically derived coherence metrics. Assumptions for the analysis were met: Homoscedasticity of residuals was given, as shown by the scatterplot of residuals, and a Kolmogorov-Smirnov test indicated normality of residuals (*D* = .112, *p* = .2). Multinomial logistic regression analysis was conducted to test whether coherence measures could predict group membership of participants and to calculate odds ratios (OR) for coherence measures. The healthy controls group was used as the reference category. Variance inflation factors indicated that multicollinearity of *z*-standardized predictor variables was likely not an issue (VIF 1.09–1.48). Three separate analyses were performed across all three groups, first entering automatically derived coherence metrics as a predictor variable, second automatically derived coherence metrics, and repetitions as predictor variables and their interaction term ACM*repetition. The third, full model was further supplemented with variables that were first, theoretically associated with coherence in NAP [see 2.4. (4–6)], and second, significantly differed between groups (see **Table 3**): differentiating markers of cohesion (LIWC), referential abnormalities, and neologisms.

## Results

### Sample Characteristics

Patients and HC did not differ significantly regarding age and verbal IQ. Patients with and without signs of positive FTD did not differ significantly regarding diagnosis, duration of illness, or current medication. Patients with positive FTD were more often male, inpatients, and rated to be overall more severely ill than those without positive FTD. Patients with positive FTD also had higher ratings for various other symptoms than patients without ratings of positive FTD (**Table 1**). None of the sample characteristics that differed significantly between groups – i.e. differences in sex, patient status, overall severity of illness as well as severity of symptoms other than positive FTD – were significantly associated with differences in automatically derived coherence metrics.

### NET Interviews

**Table 2** shows that interview length and word count differed significantly between groups: Patients with positive FTD had longer interviews and used more words than healthy controls. Patients without positive FTD had shorter interviews and used fewer words than healthy controls. This difference persisted after cleaning transcripts of unknown words (i.e. words that were not assigned vectors in the GloVe model), verbal fillers, and sentences only containing stop words. The number of removed words did not differ significantly between groups.

### Coherence Analysis

#### Predicting Clinical Ratings With Automatically Derived Coherence Metrics

**Figure 2:**
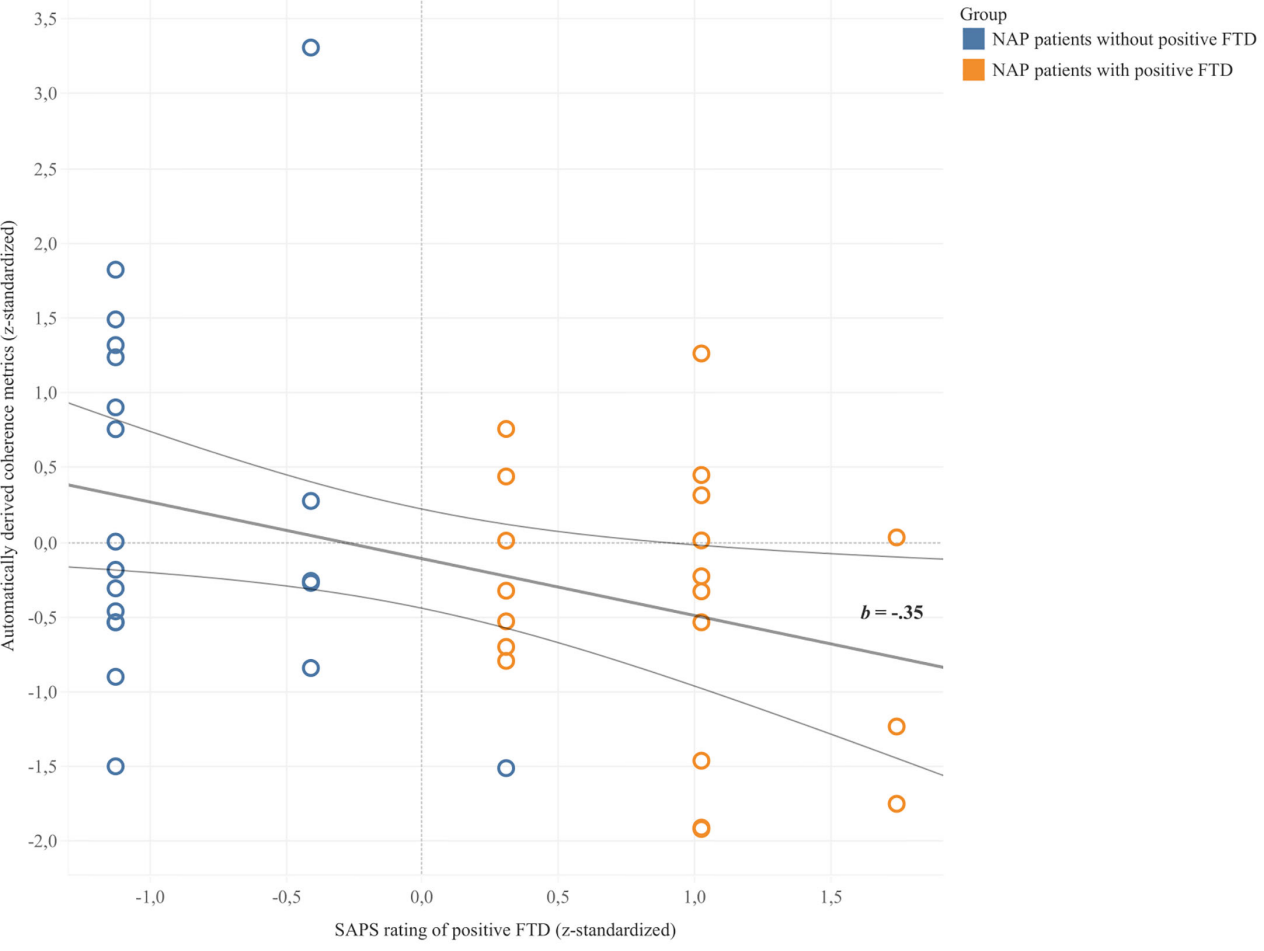
Linear regression between z-standardized values of automatically derived coherence metrics and clinical SAPS ratings of positive FTD. Trend line *b* depicted with 95% confidence bands.

Coherence metrics from automatic coherence analysis significantly predicted clinical ratings of positive FTD in NAP patients (*b* = -.35, 95% CI [-.6, -.04], *p* = .028) in a linear regression model. 9.8% of the variance in clinical ratings was explained by automatically derived coherence metrics (*F* = 5.23, *p* = .028). As illustrated in **Figure 2**, automatically derived coherence metrics appeared to be lower for patients with ratings of positive FTD and higher for those without ratings of positive FTD. Results did not change when excluding the outlier value in the group without positive FTD.

#### Predicting Group Membership With Automatically Derived Coherence Metrics

**Table 4** summarizes the results of the multinomial logistic regression analysis while **Table 5** shows the model fitting criteria and tests of significance. The first model, which only included automatically derived coherence metrics as an independent variable, was not significant, while the second model obtained a significant improvement over the intercept-only model. Automatically derived coherence metrics significantly predicted group membership for patients with positive FTD as compared to healthy controls, while inadequate repetitions significantly predicted group membership for patients without positive FTD as compared to healthy controls. The interaction effect of the independent variables was non-significant. Model 2 correctly classified 25% of healthy controls, 60% of patients with positive FTD, and 50% of patients without positive FTD.

**Table 4:** Prediction of group membership: results of three multinomial logistic regression analysis models.

	NAP patients without positive FTD				NAP patients with positive FTD			
	*b*	SE	*p*	OR [95% CI]	*b*	SE	*p*	OR [95% CI]
Model 1								
Constant	.004	.323	.990		−.089	.339	.792	
ACM	−.020	.318	.950	.980 [.526–1.828]	−.769	.382	.044	.464 [.219–.979]
Model 2								
Constant	−.167	.369	.651		−.148	.370	.690	
ACM	.031	.353	.930	1.032 [.517–2.058]	−.946	.434	.029	.388 [.166–.909]
Repetitions	.938	.418	.025	2.555 [1.126–5.799]	−.227	.418	.588	.797 [.351–1.809]
ACM*Rep	−.520	.425	.221	.594 [.258–1.367]	−.406	.417	.330	.666 [.294–1.508]
Model 3								
Constant	1.124	.763	.141		.918	.773	.235	
ACM	.230	.378	.543	1.259 [.599–2.642]	−.235	.522	.653	.791 [.284–2.200]
Repetitions	.852	.422	.044	2.345 [1.025–5.367]	−.493	.530	.353	.611 [.216–1.728]
Neologisms	2.152	1.22	.078	8.603 [.787–94.053]	2.658	1.25	.033	14.27 1.239–164.27]
Ambig. ref.	1.318	1.01	.192	3.737 [.516–27.069]	2.485	1.04	.017	12.00 [1.560–92.238]
Differentiat.	−.282	.398	.479	.754 [.346–1.644]	−1.14	.514	.026	.319 [.116–.872]

The reference category is healthy controls.

ACM, automatically derived coherence metrics; FTD, formal thought disorder; NAP, non-affective psychosis.

**Table 5:** Model evaluation for the multinomial logistic regression analysis.

	Model fitting criteria		Likelihood ratio tests	
	AIC	Nagelkerke *R^2^*	*χ²*	*p*
Model 1	134.025	.104	5.808	.055
Model 2	130.936	.276	16.897	.010
Model 3	112.867	.575	42.966	.000

AIC, Akaike Information Criteria.

#### Full Multinomial Logistic Regression Model

As shown in **Table 5**, testing the third full model versus an intercept-only model was statistically significant. The model correctly classified 75% of healthy controls, 70% of NAP patients with positive FTD, and 50% of NAP patients without positive FTD. All predictor variables except automatically derived coherence metrics were significant in classifying patients with positive FTD as compared to healthy controls. In the patient group with positive FTD, the probability of LIWC cohesive differentiation markers was lower as compared to healthy controls, while the use of neologisms and ambiguous referents was more likely to be found among patients with positive FTD as compared to healthy controls. **Table 4** also reveals that high numbers of, i.e. inadequate, repetitions significantly predicted membership in the patient group without positive FTD as compared to healthy controls, which was not the case for patients with positive FTD. As shown in **Table 5**, Model 3 received a lower Akaike Information Criteria (AIC) score than the other two models indicating the best model fit.

## Discussion

This pilot study tested a computational linguistic approach to modeling coherence in the free speech of patients with non-affective psychosis. Modeling of coherence followed the *Incoherence model* by Bedi et al. (12), and used TF-IDF sentence embeddings and GloVe word embeddings. Results from linear regression analysis seem to support Hypothesis 1 that automatically derived coherence metrics match clinical ratings of positive FTD: NAP patients with higher ratings of positive FTD displayed lower automatically derived coherence metrics. These results agree with findings in our preliminary study (14) and former research in this field (11, 13). The small percentage of explained variance in clinical ratings of positive FTD indicates that automatically derived coherence metrics may not be sufficient in predicting incoherence in NAP patients. This is also suggested by insufficient prediction of Model 1 in multinomial logistic regression analysis which only included automatically derived coherence metrics as the independent variable.

Hypothesis 2 postulated an improved prediction of group membership by automatically derived coherence metrics when controlling for bias by inadequate repetition. We therefore integrated an automated assessment of inadequate repetition of emotion keywords to regression model 2, which was significant. Results indicated that automatically derived coherence metrics, not repetitions, predicted group membership in the patient sample with positive FTD, while the reverse pattern was found for the patient sample without positive FTD: repetitions predicted status as a patient, while coherence metrics appeared similar to healthy controls. In other words, patients without FTD seemed to hold on to the literal repetition of the concept at hand, while thought-disordered patients derailed from the conversation topic and jumped to semantically more distant associations. One possible explanation could be that verbal perseveration, e.g. as a catatonic symptom, indicates associative restraint rather than associative loosening, and is not included in SAPS criteria for FTD. Moreover, repetitions might suggest a failure to change mental sets flexibly, and may hence indicate executive dysfunction, which might be even more prominent in patients without striking positive symptoms (55–57). Results show that, on the one hand, including repetitions in automated coherence analysis improved prediction. On the other hand, due to non-significant interaction effects, automatically derived coherence metrics did not seem to be overestimated by repetitions. However, our measure of repetitions can only serve as an approximation of perseveration in a closely specified context, probably missing other causes of inadequate repetition. Future studies with more complete automated measures of perseveration may find significant interaction effects.

We found support for Hypothesis 3 that modeling disordered thought can be improved by integrating other quantifiable coherence measures: classification of groups was improved in the full multinomial logistic regression model. Interestingly, when additional FTD measures like referential abnormalities, neologisms, and cohesive markers were included as predictors, automatically derived coherence metrics were no longer a significant predictor of diagnostic status (Model 3). One possible explanation could be that disordered thought (based on clinical ratings of global positive FTD and incoherence or tangentiality) was better predicted by referential abnormalities, cohesion markers, and neologisms because they were associated with the listener’s subjective impression of “incoherent” speech. Ambiguous referents and neologisms may lead to unintelligibility for the listener, which in turn biases their clinical rating of associative loosening. In contrast, the mere occurrence of remote semantic relations in the patients’ speech, represented by lower automatically derived coherence metrics, could still be tolerated by trained clinicians and not perceived as overly confusing. Therefore, linguistic incoherence alone might not be the decisive criterion for clinical ratings of FTD. This signals different conceptualizations of coherence – either as a “product of psychological representations” [(53), p. 193f.] formed by the listener or as an inherent feature of the text. This may hint at the potentially unique contribution of automatically derived coherence metrics to clinical research. They aim to represent impairments in coherence inherent in the speaker’s speech and thus, capture characteristics of incoherent discourse that might not be detectable by clinicians. This may explain why Bedi et al. (12) and Corcoran et al. (15) found automatically derived coherence metrics superior to clinical impression in the prediction of psychosis development in high-risk individuals, who do not show obvious neologisms or referential abnormalities. Thus, our results call for further research in sub-clinical samples to corroborate the value of automated coherence analysis.

Alternative FTD measures that markedly improved the full multinomial logistic regression model were referential abnormalities, neologisms, and LIWC cohesive differentiating markers (if, when, but, although, etc.). The fact that referential abnormalities were more likely in the thought-disordered patient sample than in the other two groups is in line with findings by Docherty, Cohen, Nienow, Dinzeo, and Dangelmaier (58), Docherty (59), and Rochester and Martin (1). According to the latter authors, referential abnormalities might emerge when patients fail to perpetuate meaning across sentences, i.e. to maintain semantic coherence. Neologisms can result from reduced adherence to pragmatic rules resulting in violation of conventional word usage (60). A higher probability of neologisms for patients with high ratings of positive FTD can be a sign of incoherent “schizophasia” (38). Also, LIWC cohesive differentiating markers predicted patient status. It should be noted that other LIWC categories linked to cohesion did not differ significantly between groups, i.e. syntactic cohesion seemed to be relatively intact in the patient sample. One could argue that the LIWC category *differentiation* represents a relatively complex relation between discourse units where opposing concepts and their association need to be maintained concurrently. Thus, one could speculate that it was more error-prone than other relations.

One limitation of this pilot study is the representational quality of the trained model. Our model was trained on the German version of Wikipedia and may be inferior to models trained in English [see (11–13)]. In Iter et al. (13) for instance, who trained their models on the respective English Wikipedia dump, training data nearly triples German training data. Since German models need to generalize over a wider morphological spread, there is an even greater need for larger amounts of training data^2^. Moreover, especially multinomial logistic regression results should be carefully interpreted due to the relatively small sample size and require replication. Large confidence intervals of odds ratios (**Table 4**) indicate the uncertainty of analysis. We therefore conducted a post-hoc power analysis for the linear regression analysis and ANOVA for ACM–our most relevant predictor (power of ANOVAs for other predictors was larger than .80). While the power analysis for linear regression was sufficient (power of linear regression: 0.862, effect size f²: 0.127), power for ANOVA (ACM) was small with 0.586, effect size f: 0.325. If expecting an effect of .325, a sample size of at least *n* = 96 would be needed to achieve a power of .80 and could be recommended to future studies. Moreover, the selection of variables that had been demonstrated to distinguish patient groups in this sample of cases might have produced a bias in favor of the multinomial logistic regression model. Also, the reliability of interpretations could be improved by balancing patient samples for defining characteristics. Another interesting question for future studies may be whether automatically derived coherence metrics can predict other measures of FTD, e.g. self-report instruments such as the rating scale for the assessment of objective and subjective formal Thought and Language Disorder (61).

In summary, automated coherence analysis can serve as an objective measure of concept overlap, capturing inherent features of incoherent speech that are independent from the listener’s impression. Nevertheless, clinical evaluations of coherence are not only informed by concept similarity but also comprehensibility, which was impaired by factors such as referential abnormalities and neologisms. It can be assumed that linguistic parameters of coherence will enrich assessments of FTD, but that several facets leading to the clinical diagnosis of FTD, as well as parameters potentially biasing automated coherence metrics, have to be equally considered. The study shows that interdisciplinary collaboration between computational linguistics and psychiatry can enable mutual stimulation and further conceptual understanding.

## Data Availability Statement

The raw data supporting the conclusions of this article will be made available by the authors, without undue reservation.

## Ethics Statement

The studies involving human participants were reviewed and approved by Ethikkommission der Charité – Universitätsmedizin Berlin. The patients/participants provided their written informed consent to participate in this study.

## Author Contributions

SJ and EH conceptualized and designed the study, conducted the analysis, and wrote the first draft. All authors contributed to the article and approved the submitted version.

## Funding

We acknowledge support from the German Research Foundation (DFG) and the Open Access Publication Fund of Charité – Universitätsmedizin Berlin.

## Conflict of Interest

The authors declare that the research was conducted in the absence of any commercial or financial relationships that could be construed as a potential conflict of interest.

## Footnotes

1 In order to prevent artifacts of the reference corpus, such as e.g. misspellings, to accidentally influence the derived weights, we required a word to have appeared in at least two documents for it to be included in the statistics informing the TF-IDF-measure. The highest possible weight thus is the number of appearances of a word within the sentence under consideration divided by two. 

2 Since von Schlegel (55), languages are located along a continuum of morphological complexity. German is of a more synthetic type than English, which lies closer to the analytical end of the spectrum. This notion reflects that German exhibits more inflectional morphology, such as e.g. case-marking suffixes, than English, which predominantly uses positional means or prepositions to mark case. Semantically rich words (nouns, verbs, adjectives) hence encompass larger sets of surface forms in the German than the English training data.
